# Supplementary material for: Learning curve and surgical outcome of robotic assisted colorectal surgery with ERAS program
Source: Sci Rep. 2022 Nov 29;12:20566. doi: 10.1038/s41598-022-24665-w (PMC9709162; doi:10.1038/s41598-022-24665-w)
Supplement: Supplementary file 1 — Supplementary Information 1. [file 41598_2022_24665_MOESM1_ESM.zip › LSC Raw data-Ñ[▒K/053/HS_ñw╝╨░O▒Kñσ.pdf]

正式摘要(電子簽章：000000132315893)

W55 / W55 ☐ 外文標題(注意:無英文姓名-請補病人基本檔!) ☐【讀取】憑證

臺中榮民總醫院

出院病歷摘要

轉入號：　　　轉出號：

[redacted]

| | |
|---|---|
| (8)流水編號 | |
| (9)入院日期 | 108/10/22 (2019) 直腸外科 W55 - 263 |
| (10)轉科日期 | |
| (11)出院日期 | 108/12/19 (2019) 住院天數計 58 日 |

## 診斷

- 入院 :1. Distal T-colon cancer with obstruction, s/p T-loop colostomy and tumor biopsy
- 出院 :1. Distal T-colon cancer with bowel obstruction, cT3N0M1a, stage 4A, s/p T-loop colostomy s/p ext. right hemicolectomy with D3 disection on 2019/11/6 pT4aN1a(1/32)M1a(lung?)
  2. Gout attack
  3. Urosepsis, ureter stone related post Right PCN
  4. AKI, KDIGO stage 2, r/i infection, obstructive uropathy, medication related ATN
  5. Hyperglycemia
  6. Non-AG metabolic acidosis (AG=11.8, Delta AG=0.2, UAG=41.8)
  7. GB stone s/p LC 2019/11/6

## 主訴

- Abdominal fullness, anroexia, and general weakness for 3+ days.

## 病史

- This 68 y/o male has the underlying dusease of gout.
  He complained diffuse abaominal discomfort and fullness sensation for days. He described the abdominal discomfort as cramping pain on and off, sudden attack and relived after 5 minutes, and the intensity was geeting stronger in days. He had epigastralgia, nausea, and decreased appetite. There was no vomiting, change of bowel habit, or body weight loss. He denied fever, URI symptoms, palpitation, or heartburn sensation.
  He visited 大千 Hosp. ED for the progressive and persistent diffuse discomfort. Abdominal CT revealed colon tumor at spleenic flexure, complicated with ileus. He was transferred to VGHTC for further evaluation under his family's will.
  T-loop colostomy and tumor biopsy was performed under the impression of Distal T-colon Ca with obstruction on 10/21. After the surgery, he was admitted for a through tumor survey.

## 體檢發現

- 
  - General appearance :
    - acute ill looking , conscious clear , without cardiopulmonary distress
    - Vital signs自動帶入date 體溫(BT) 脈搏(P) 呼吸(R) 血壓(BP) 身高(BH) 體重(BW) BMI
      20191022 37.5℃ 115次/分 18次/分 180/62mmHg 175cm 60kg 19.6
    - Vital signs: B.P. 180/62 mmHg , P.R. 115 /min , R.R. 18 /min , B.T. 37.5 ℃
  - HEENT :
    - Eyes: no anemic conjunctiva, no icteric sclera, normal EOM
    - ENT: no otorrhea, no rhinorrhea, no erythematous mucosa, no injected tonsil, no deformity
  - Neck: supple, freely movable, trachea midline,no deformity, no engorged jugular vein , no palpable thyroid or lymph node enlargement
  - Chest:
    - Inspection: Grossly normal, symmetric and fully expansion, no accessory muscle used, no operation scar, no spider angiomata, no gynecomastia
    - Palpation: no palpable mass or nodule, no subcutaneous emphysema, no tenderness
    - Ausculation: clear breathing sound
  - Heart:
    - Palpation: no thrill, no heave
    - Percussion: normal heart size
    - Ausculation: regular heart beat, normal S1, S2, no gallop, no murmur
  - Extremities: No deformity, full ranged of motion without limitation, no clubbing finger or nail cyanosis, no pitting edema, no ulcer or pigmentation, no obvious skin lesion, no varicose vein, no bone, joint or muscle tenderness

  **CRS CONDITION**

  - Karnofsky performance status: (100) Normal, no complaints, no evidence of disease
  - PE of Abdomen:
  - __1. Inspection: s/p colostomy
  - __2. Ausculation: normal bowel sound
  - __3. Palapation: no tenderness
  - __4. Percussion: dullness
  - CEA: 39.04 ng/ml
  - Imaging Studies:
  - Colonoscope report: nil
  - Sono , CT of abdomen and chest Films:檢驗項目:CHEST PA VIEW 報告日期:1081025
    主 訴：
    Result:
    Finding:
    Chest and KUB:
    1. Increased intestinal and colon gas are noted diffusely over the abdomen. R/O intestinal obstruction. Suggest follow up closely.
    2. DJD change along L-spine.
    3. Radiopaque gall stone over right upper abdomen. An ovoid calcification over right paravertebral region at L3 level. R/O ureteral stone.
    4. Mild cardiomegaly and tortuosity along the T-aorta.R/O hypertension.
    5. Chronic interstitial infiltration in bil. lower lungs.
    Impression:
    As above description.
    檢驗項目:ABDOMEN (KUB),SUPINE 報告日期:1081025
    主 訴：
    Result:
    Finding:
    Chest and KUB:
    1. Increased intestinal and colon gas are noted diffusely over the abdomen. R/O intestinal obstruction. Suggest follow up closely.
    2. DJD change along L-spine.
    3. Radiopaque gall stone over right upper abdomen. An ovoid calcification over right paravertebral region at L3 level. R/O ureteral stone.
    4. Mild cardiomegaly and tortuosity along the T-aorta.R/O hypertension.
    5. Chronic interstitial infiltration in bil. lower lungs.
    Impression:
    As above description.
  - Figures:
    1. colon & rectum

## 手術日期及方法

- 手術日期及方法20191106 1. Laparoscopic right hemicolectomy with side-to-side anastomosis, GIA 80/3.8 *2, re-inforcement with silk (+).

- 2. Vessel ligation : ileo-colic, right colic, middle colic vessels and left colic artery.
  3. Cut margin: proximal (ileum) 5 cm, (colon) 30 cm, distal 15 cm.
  4. Repair of mesentery defect: nil.
  5. Laparoscopic cholecystectomy
  6. Take down the T-colostomy
  7. Two 12mm penrose drain at Morrison's pouch and splenic fossa.
  20191105 Laparoscopic Cholecystectomy
  20191022 Loop colostomy
  Intraoperative colonoscopy for biopsy

---

## 住院治療經過

- After admission, we arranged tumor survey. Blood was obtained tested, and showed elevated CEA 39.04 ng/mL, and CA199 1381U/mL. The AFP level was within normal range. Chest CT was done on 10/24, and revealed nodules at lung (RLL and RUL), a liver nodule at S4, an osteoblastic nodules at T12 veterbral body, and a group low attenuation nodules at left paracolic region, metastasis could not be excluded. Liver MRI was done on 10/27, and showed several nodules over both lobes of liver, hemangiomas was considered first. Whole body PET scan was done on 10/30, and C/W T-colon cancer, and there was no increased FDG uptake in liver. We consulted GS Dr. 吳峰圳 for the liver tumors, and tumor resection was not indicated due to there was no evidence of malignancy of the liver tumor on PET scan. Laparoscopic right colectomy and anastomosis was done on 11/5. Post operation, patient was under stable condition.Whole body PET scan revealed 1.left upper abdomen (intestine and/or pericolonic LNs) may be due to tumor involvement (Grade 4). 2.A small nodule at the right lower lung without FDG uptake. Tumor involvement can't be ruled-out. 3. The mildly increased FDG uptake at the abdominal wall (colostomy)(Grade 1). 4.The mildly increased FDG uptake at the right axillary LNs, the lateral aspect of right buttock may be due to inflammation or infection (Grade 1). However, patient was noted of whitesh discharge from Penrose on 11/9, ascites revealed TG:399,R/O chile leak . We change NPO to no oil diet on 11/11 due to clear light yellowish liquid discharge from penrose.But patient started to suffer abdominal colic pain after meal so we changed to try water for bowel rest today and keep current treatment.Patient also complained of left ankle dorsiflextion pain and Ketorolac, Colchicine were administered on 11/9. However, he complained of right wrist pain on 11/10 so we consulted AIR and metholone was administered for Gout acute attact . Patient complained of increased nocturia without fever or dysuria..Urine culture revealed pseudomonas aergunosa so we shifted antibiotics to cefepime.Due to occasional clear yellowish fluid mixed with some whitish clot from penrose, changed NPO to non-oil soft diet and adequate hydration on 11/17. General weakness and left penrose pus was noted on 11/23. LAB data revealed AKI r/o dehydration and sepsis. We arranged for abdominal CT and it showed right hydronephrosis and ileus. Foley inserted and NG put in for decompression. Metabolic acidosis was suspected and antibiotic and sodium bicarbonate was giving. The patient started to have conscioulness drowsy since 11/23, lab data revealed servere metabolic acisosis and deteriotate renal function , CT scan was done to rule out intraabdminal infection, and it showed no significant abcess howervr right hydronephrosis was noted comparing to last CT. Urine routine also showed pyuria. Under the impression of urosepsis, PCN was arrange. After PCN the patient GCS still around E3V2M5, due to persisted metabolic acisosis, we consult NEPHRO doctor and they suggest no indications for emergent HD. Therfore standing sodium bicarbonate was used for the patient and keep cefepime for the patient. In the morning of 11/25 due to agitate of the patient, ETT was done and transfer to SICU. At SICU, Ceftazidime and hydration were given for his urosepsis control. We stopped NSAID usage for suspected AKI related to medication. Non-AG metabolic acidosis (AG=11.8, Delta AG=0.2, UAG=41.8) was considered after consulting NEPHRO, therefore, alkali therapy with NaHCO3 supplement was arranged since 11/25. KCl injection was kept for hypokalemia. His consciousness was drowsy at first. We administered Flumazenil and stopped Precedex and Propofol on 11/28. His consciousness was clear without fever noted on 12/2. CXR showed RML atelectasis on 12/3, and he was under spirometer coach training. Endotracheal tube was removed on 12/2 and foley's catheter and NG tube were removed on 12/3. Under his stable condition, he was transfered to ordinary ward for further management. His bil. Penrose was removed on 12/6. He is now under soft diet, and no discomfort was complained. He had fever to 38.3 on 12/6 with R't hand MCP joint reddish and swelling suspected gout attack, thus NSAID and colchicine were prescribed. Uric acid was in normal range. Fever survey was done. U/A was pyuria and U/C was no becteria. Sputum culture showed normal flora. His CXR showed increased insterstitial infiltration over bilateral lung fields and patchy consolidation over right lower lung, r/o pneumonia. Therefore, Ceftazidime and Metronidazol was used(12/6-12/13). He recovered well after this treatment and his vital sign was stable recently. However, he had hyperkalemia(K:6.1) on 12/17, but without any clinical symptom and discomfort. His EKG showed sinus rhythm. Kalimate powder was givan for eliminating K+ and NSAID stop using. His potassium was declined to normal range on 12/18. Under stable condition, he discharge on 12/19 and will stay in nursing home.

---

## 合併症

---

## 一般檢查

- 累積報告：

| DATE | TIME | ALB | TP | BIL.T | BIL.D | ALKP | AST | ALT | LDH | CHOL | TG | HDL-C | LDL | UA | NH3 | AMY |
|---|---|---|---|---|---|---|---|---|---|---|---|---|---|---|---|---|
| 1081021 | 2227 | 4.1 | 7.3 | 0.8 | | 77 | | 14 | 202 | | | | | | | 72 |
| 1081028 | 0943 | 3.3 | 6.1 | 0.19 | 0.10 | 55 | 34 | 7 | 215 | | | | | | | |
| 1081028 | 1513 | | | | | | | | | 45 | 115 | | | | | |
| 1081126 | 0546 | | | | | | | | | | | | | 6.1 | | |
| 1081126 | 1145 | | | 0.22 | 0.10 | | 14 | 8 | | | | | | | | |
| 1081128 | 0505 | | | | | | | | | | | | | | 61 | |
| 1081128 | 0507 | | | 0.20 | 0.11 | 101 | 12 | 7 | | | | | | | | |
| 1081202 | 0555 | 2.7 | | 0.14 | 0.09 | | 15 | 6 | 199 | | | | | | | |
| 1081205 | 0920 | 4.1 | | 0.46 | | 101 | | 7 | | | | | | | | |
| 1081207 | 0909 | | | | | | | | | | | | | 5.1 | | |
| 1081209 | 1017 | 3.5 | 6.5 | 0.23 | 0.12 | 87 | 15 | 6 | 169 | | | | | | | |
| 1081212 | 0916 | 3.5 | | 0.15 | 0.09 | | 19 | 7 | | | | | | | | |
| 1081217 | 0916 | 4.1 | | 0.30 | | | | 15 | | | | | | | | |

| DATE | TIME | WBC | RBC | HGB | HCT | MCV | PLT | BLST | NEUT | BAND | SEG | LYM | MON | EOS | BAS |
|---|---|---|---|---|---|---|---|---|---|---|---|---|---|---|---|
| 1081021 | 2227 | 7140 | | 9.5 | | | | | 76.3 | | | 13.4 | 9.4 | 0.6 | 0.3 |
| 1081023 | 0909 | 14250 | 4.56 | 9.0 | 30.2 | 66.2 | 309 | | 84.7 | | | 8.4 | 6.7 | 0.1 | 0.1 |
| 1081125 | 1632 | 11900 | 3.47 | 8.6 | 25.6 | 73.8 | 231 | | 88.0 | | | 6.7 | 3.9 | 1.1 | 0.3 |
| 1081126 | 1134 | 9020 | | 8.1 | | | 199 | | | | 80.0 | 12.0 | 5.0 | 3.0 | 0.0 |
| 1081128 | 0504 | 8390 | 4.19 | 10.5 | 32.5 | 77.6 | 164 | | 80.2 | | | 10.0 | 4.9 | 4.4 | 0.5 |
| 1081202 | 0535 | 5730 | 3.86 | 10.0 | 30.5 | 79.0 | 170 | | | | 78 | 12 | 2 | 8 | |
| 1081205 | 0852 | 9930 | 4.68 | 12.1 | | | 332 | | 78.4 | | | 14.7 | 5.6 | 0.9 | 0.4 |
| 1081206 | 1347 | 17200 | 4.72 | 11.9 | | | 396 | | | 2.0 | 89.0 | 7.0 | 2.0 | 0.0 | 0.0 |
| 1081209 | 1019 | 6300 | | 10.1 | | | 454 | | 71.9 | | | 16.3 | 6.2 | 4.6 | 1.0 |
| 1081212 | 0904 | 5830 | 4.01 | 10.4 | 33.1 | 82.5 | 378 | | 55.8 | | | 32.2 | 7.7 | 2.9 | 1.4 |
| 1081217 | 0851 | 7980 | 4.39 | 11.4 | | | 356 | | 49.5 | | | 41.7 | 5.0 | 2.8 | 1.0 |

| DATE | TIME | NA | K | CL | CA | BUN | CREAT | Mg | P | CRP | ESR | PCT | LIPASE | LACTATE |
|---|---|---|---|---|---|---|---|---|---|---|---|---|---|---|
| 1081021 | 2227 | 135 | 4.4 | | 8.3 | 23 | 1.50 | | | 2.305 | | | | |
| 1081021 | 2228 | | | | | | | | | | | | | 8.4 |
| 1081125 | 1016 | 151 | 3.1 | 122 | 8.7 | 52 | 4.17 | | | | | | | |
| 1081125 | 1632 | 149 | 3.0 | | | | 4.19 | | | | | | | |
| 1081125 | 2220 | | 3.3 | | | | | | | | | | | |
| 1081126 | 0546 | | 3.0 | | | | | | | | | | | |
| 1081126 | 0946 | | | | | | | 1.8 | 3.1 | | | | | |
| 1081126 | 1126 | | | | | | | | | | | | | 5.4 |
| 1081126 | 1145 | 148 | 3.3 | | | 48 | 3.91 | | | | | | | |
| 1081126 | 1629 | | 3.5 | | | | | | | | | | | |
| 1081126 | 2313 | | 3.3 | | | | | | | | | | | |
| 1081127 | 0536 | | 3.2 | | | | | | | | | | | |
| 1081127 | 1526 | | 3.4 | | | | | | | | | | | |
| 1081127 | 2257 | | 3.5 | | | | | | | | | | | |
| 1081128 | 0507 | 140 | 3.5 | 109 | 8.2 | 39 | 3.66 | | | | | | | |
| 1081128 | 1520 | | 3.4 | | | | | | | | | | | |
| 1081128 | 2347 | | 3.6 | | | | | | | | | | | |
| 1081129 | 0533 | | 3.5 | | | | | | | | | | | |
| 1081129 | 1621 | | 3.7 | | | | | | | | | | | |
| 1081129 | 2232 | | 3.9 | | | | | | | | | | | |
| 1081130 | 0458 | | 3.6 | | | | | | | | | | | |
| 1081201 | 0519 | | 4.1 | | | | | | | | | | | |
| 1081202 | 0540 | | | | | | | | | | | | | 9.0 |
| 1081202 | 0555 | 134 | 4.3 | 103 | 7.6 | 24 | 2.25 | 1.5 | 2.9 | | | | | |
| 1081203 | 0505 | | 4.0 | | | | | | | | | | | |
| 1081205 | 0920 | 137 | 4.3 | 101 | 8.9 | 19 | 1.61 | | | | | | | |
| 1081206 | 1333 | | | | | | | | | | | | | 16.5 |
| 1081207 | 0909 | 135 | 3.8 | 101 | | 21 | 1.67 | | | | | | | |
| 1081209 | 0958 | | | | | | | | | | | | | 14.7 |
| 1081209 | 1017 | 136 | 4.0 | 105 | 8.4 | 25 | 1.62 | | | | | | | |
| 1081212 | 0916 | 139 | 4.5 | | 8.7 | 22 | 1.35 | | | | | | | |
| 1081217 | 0916 | 137 | 6.1 | 107 | 9.7 | 19 | 1.08 | | | | | | | |
| 1081217 | 1308 | | 5.3 | | | | | | | | | | | |
| 1081218 | 0909 | | 5.5 | | | | | | | | | | | |

| DATE | TIME | GLUCOSE | PAB | TRF | ZN | CU | KETONE | CK | CKMB | PT-P | PT-C | APTT-P | APTT-C | TRO-T |
|---|---|---|---|---|---|---|---|---|---|---|---|---|---|---|
| 1081021 | 2227 | 122 | | | | | | 60 | | 11.3 | 10.1 | 25.1 | | |
| 1081021 | 2228 | | | | | | | | | | | | | 10.79 |
| 1081123 | 1652 | 157 | | | | | | | | | | | | |

| DATE | TIME | SP-GR | PH | PROT | GLUC | KETON | BILI | UROB | OB | NITR | LEU | RBC | WBC | EPCEL |
|---|---|---|---|---|---|---|---|---|---|---|---|---|---|---|
| 1081022 | 0123 | 1.021 | 6.0 | 1+ | 500.0 | 2+ | Negative | 0.2 | Negative | Negative | 1+ | 0-2 | 35-50 | 0-1 |

| | | | | | | | | | | | | | | |
|---|---|---|---|---|---|---|---|---|---|---|---|---|---|---|
| 1081023 | 0915 | 1.014 | 6.0 | 1+ | 250.0 | Trace | Negative | 0.2 | Trace | Negative | 1+ | 2-5 | 2+ | 0-1 |
| 1081114 | 1238 | 1.016 | 6.0 | 1+ | 250.0 | Negative | Negative | 0.2 | Trace | Negative | 3+ | 10-20 | 3+ | 1-2 |
| 1081123 | 2229 | 1.010 | 5.5 | 1+ | 100.0 | Negative | Negative | 0.2 | 2+ | Positive | 3+ | 0-2 | 3+ | 2-4 |
| 1081125 | 1633 | 1.009 | 6.0 | 1+ | 100.0 | Negative | Negative | 0.2 | 2+ | Negative | 3+ | 2-5 | 35-50 | 0-1 |
| 1081206 | 1427 | 1.012 | 7.0 | 2+ | >1000.0 | Negative | Negative | 0.2 | 2+ | Negative | 2+ | 2+ | + | 1-2 |
| 1081206 | 1427 | 1.012 | 6.5 | 1+ | >1000.0 | Negative | Negative | 0.2 | Trace | Negative | Trace | 2-5 | 10-20 | 0-1 |

| DATE | TIME | STOOL-OB | FAT | RBC | WBC | PARA |
|---|---|---|---|---|---|---|
| 1081029 | 0241 | Negative | | - | - | Not Found |
| 1081217 | 1659 | | | - | - | Not Found |

| DATE | TIME | PH | PCO2 | PO2 | SO2 | BEB | HCO3 | TCO2 | HGB | FIO2 |
|---|---|---|---|---|---|---|---|---|---|---|
| 1081125 | 1010 | 7.356 | 27.3 | 334.6 | 99.9 | -8.8 | 15.0 | 15.8 | | |
| 1081125 | 1633 | 7.399 | 29.7 | 222.8 | 99.8 | -5.4 | 17.9 | 18.9 | | |
| 1081128 | 0503 | 7.336 | 36.7 | 175.5 | 99.4 | -5.9 | 19.2 | 20.3 | | |
| 1081202 | 0542 | 7.417 | 34.6 | 146.6 | 99.3 | -2.0 | 21.8 | 22.9 | | |

| DATE | TIME | PH | BEB | SCVO2 | PO2 | HCO3 | HGB | PCO2 | TCO2 |
|---|---|---|---|---|---|---|---|---|---|
| 1081021 | 2226 | 7.345 | -6.2 | 75.7 | 43.9 | 18.6 | | 34.9 | 19.7 |
| 1081123 | 1650 | 7.098 | -21.6 | 58.5 | 45.7 | 6.3 | | 20.9 | 6.9 |
| 1081124 | 0711 | 7.167 | -19.4 | 66.9 | 47.8 | 7.0 | | 19.8 | 7.6 |
| 1081124 | 1600 | 7.177 | -17.3 | 52.8 | 38.0 | 9.4 | | 26.1 | 10.2 |
| 1081124 | 2325 | 7.366 | -10.0 | 97.8 | 109.2 | 13.2 | | 23.5 | 13.9 |
| 1081209 | 1015 | 7.361 | -3.7 | 75.4 | 42.6 | 21.2 | | 38.3 | 22.4 |

- 放射線報告 :**檢驗項目 :R'T PCN 報告日期 :1081125**

主 訴：
Result:
Finding:
{Percutaneous Nephrostomy, R't} Med No: 000861301C
1. After informed consent was obtained, the patient was place under prone position.
2. The right kidney was localized under sonography, and an appropriate puncture point was selected after the depth, angle, and level to the renal calyx were determined.
3. Skin was prepped and draped in the usual sterile fashion.
4. 5 mL of 2% lidocaine was infiltrated for local anesthesia.
5. A 18G PCN needle was used to puncture the right kidney and the calyx was reached. The stylet was withdrawn slowly until urine flowed out.
6. Contrast media was injected under fluoroscopy and the collecting system was visualized. A 0.038In. guidewire was inserted along the needle sheath and into the renal pelvis.
7. A 10Fr. pigtail catheter was inserted along the guidewire and into the right renal pelvis.
8. Images were taken for documentation.

Findings:
1. Mild to moderate hydronephrosis of right kidney
2. Filling defect in right U/3 ureter, compatible with right ureteral stone

Impression:
The PCN procedure of right kidney had been performed as described above.

**檢驗項目 :CT ABDOMEN-SURVEY 報告日期 :1081125**

主 訴：
Result:
Finding:
CT scan of whole abdomen without IV contrast medium injection was performed and showed:
## Patient did not receive contrast medium administration. Tissue perfusion and vascular patency could not be evaluated and subtle lesion might be under estimated.
> Much motion artifacts and causing difficulty evaluating corresponding region.
> A case of T-colon cancer s/p extended right hemicolectomy. R/O leakage or abscess formation. For survey.
> S/P colostomy, S/P drainage tubes over bil. subphrenic regions.
> S/P cholecystectomy.
> Diffuse distension of the bowel loops, adynamic ileus should be considered.
> Fat plane stranding of peritoneum, with mild ascites. R/I peritonitis.
> A 1.3cm ureteral stone at right upper third ureter, causing moderate hydronephrosis of right kidney.
> Several low attenuation nodules in bil. lobes of liver. Suggest follow up.
> Several low attenuation nodules in bilateral kidneys, renal cysts are suspected.
> Mild bilateral perirenal fat plane stranding.
> No abnormal lymphadenopathy in retroperitoneum or pelvis.
> Bil. minimal pleural effusion.
> S/P Foley's catheter.

Impression:
1. R/I adynamic ileus and peritonitis with mild ascites.
2. Right U/3 ureteral stone with moderate hydronephrosis of right kidney.
3. For other findings, please see description.

**檢驗項目 :MRI,LIVER 報告日期 :1081029**

主 訴：
Result:
Finding:
MRI of liver pre and post iv contrast:
1. Axial & Cor. HASTE T2WI without fat-saturation
2. Axial in- and out-phase GRE phase contrast MRI
3. Axial thin-slice dynamic MRI of liver pre and post bolus iv injection of Gd-contrast agent.
4. Cor. post contrast fat-saturated GRE T1WI
5. Axial diffusion weighted imaging (DWI) of liver

Findings:
0. History of colon cancer with R/O liver metastasis for F/U.
1. Post colostomy.
2. Several nodules upto 1.5cm in size over both lobes of liver with high signal intensity in T2WI, filling in pattern in venous phase and T2 shrine through effect, R/O hemangiomas firstly.
3. Asymmetrical wall thickening about 3.9cm in length at T-colon (ser.4,no.22) with diffusion restriction & mild stranding of pericolic fat plane, C/W colon cancer.
4. Small stone about 1.3cm in size over right upper third ureter, causing moderate hydronphrosis, R/I right upper third ureteral stone.
5. several gallbladder stones
6. Small bilateral renal cysts.
7. Small cystic lesion about 2.9cm in size at sub-splenic area(ser.4,no.17). Suggest F/U.
8. pancreas & spleen are without specific abnormality.
9. No obvious para-aortic or retrocaval lymphadenopathy is noted.

Impression:
1. R/O several hemangiomas over both lobes of liver. Suggest regular F/U.
2. R/I right upper third ureteral stone with moderate hydronephrosis.
3. C/W T-colon cancer.
4. Other findings, as above description

**檢驗項目 :CT, LUNG 報告日期 :1081026**

主 訴：
Result:
Finding:
Chest CT with IV contrast shows:

- > A cace of colon cancer for tumor survey
  > A solid nodule (#Ser201 #50-3.2mm) at RLL, ground glass nodules (#24-3.8mm) at RUL(attach to vessel) and RLL(#48-3.2mm)(attach to vessel) , small lung metastasis could not be excluded. Suggest follow up
  > Focal pleural thicking over left upper and basal lung fields
  > Bilateral pleural effusion
  > A nodule with poor enhancement, 1.4 cm, S4 of liver, r/o liver metastasis
  > Multiple gallstones, with the biggest one measures 1.8 cm,
  and with mild wall thickening of fundus of GB.
  > Ascites
  > A group low attenuation nodules about 2.6 cm at left paracolic region(Ser201 #73), R/O LN metastases
  > Osteoblastic nodules at T12 veterbral body, bony metastasis could not be excluded
  > s/p colostomy at left upper abdominal region
  > Spot calcification of p-LAD.
  Impression:
  A cace of colon cancer for tumor survey
  > A solid nodule (#Ser201 #50-3.2mm) at RLL, ground glass nodules (#24-3.8mm) at RUL and RLL (#48-3.2mm), small lung metastasis could not be excluded. Suggest follow up
  > R/O liver, T12 veterbral bony metastasis and metastasis lymphadenopathy

  檢驗項目 :**CHEST PA VIEW** 報告日期 :**1081025**
  主 訴 :
  Result:
  Finding:
  Chest and KUB:
  1. Increased intestinal and colon gas are noted diffusely over the abdomen. R/O intestinal obstruction. Suggest follow up closely.
  2. DJD change along L-spine.
  3. Radiopaque gall stone over right upper abdomen. An ovoid calcification over right paravertebral region at L3 level. R/O ureteral stone.
  4. Mild cardiomegaly and tortuosity along the T-aorta.R/O hypertension.
  5. Chronic interstitial infiltration in bil. lower lungs.
  Impression:
  As above description.

  檢驗項目 :**ABDOMEN (KUB),SUPINE** 報告日期 :**1081025**
  主 訴 :
  Result:
  Finding:
  Chest and KUB:
  1. Increased intestinal and colon gas are noted diffusely over the abdomen. R/O intestinal obstruction. Suggest follow up closely.
  2. DJD change along L-spine.
  3. Radiopaque gall stone over right upper abdomen. An ovoid calcification over right paravertebral region at L3 level. R/O ureteral stone.
  4. Mild cardiomegaly and tortuosity along the T-aorta.R/O hypertension.
  5. Chronic interstitial infiltration in bil. lower lungs.
  Impression:
  As above description.

---

特殊檢查

- 檢驗項目 :**Stool Culture** 檢體:**Stool** 報告日期 :**1081219**
  陰性結果 : Neg. for Salmonella, Shigella and Vibrio.
  陽性結果 菌落數目
  Ampicillin Cefazolin ClindamycinClarithromyGentamicin Amikacin Chloromycin
  (1)
  (2)
  (3)
  (4)
  Unasyn Penicillin GM(120ug) SMX+TMP Vancomycin Oxacillin Cefpirome
  (1)
  (2)
  (3)
  (4)
  Carbapen(IPM)CefoperazonCefotaxime Ceftazidime Cefepime Ceftriaxone Flomoxef
  (1)
  (2)
  (3)
  (4)
  MetronidazoleCiprofloxacMinocyclineTeicoplanin Tazocin CefmetazoneAztreonam
  (1)
  (2)
  (3)
  (4)
  Tigecycline Amox./Clav.Cefoxitin Levofloxa.Carbapen(MPM) Erythromycin
  (1)
  (2)
  (3)
  (4)
  Moxifloxacin Linezolid Ertapenem
  (1)
  (2)
  (3)
  (4)

  檢驗項目 :**Bacterial Culture(Ordinary)** 檢體:**Sputum** 報告日期 :**1081211**
  陰性結果 : Normal mixed flora presented.
  陽性結果 菌落數目
  Ampicillin Cefazolin ClindamycinClarithromyGentamicin Amikacin Chloromycin
  (1)
  (2)
  (3)
  (4)
  Unasyn Penicillin GM(120ug) SMX+TMP Vancomycin Oxacillin Cefpirome
  (1)
  (2)
  (3)
  (4)
  Carbapen(IPM)CefoperazonCefotaxime Ceftazidime Cefepime Ceftriaxone Flomoxef
  (1)
  (2)
  (3)
  (4)
  MetronidazoleCiprofloxacMinocyclineTeicoplanin Tazocin CefmetazoneAztreonam
  (1)
  (2)

- (3)
  (4)
  Tigecycline Amox./Clav.Cefoxitin Levofloxa.Carbapen(MPM) Erythromycin
  (1)
  (2)
  (3)
  (4)
  Moxifloxacin Linezolid Ertapenem
  (1)
  (2)
  (3)
  (4)

  **檢驗項目:GRAM STAIN 檢體:Sputum 報告日期:1081209**
  1) PMN >25 /LPF
  2) Squamous epithelial cell 25 /LPF
  3) Few Gram postive bacilli
  4) Few Gram postive cocci in group
  5) Few Gram negative cocci
  @ SPUTUM: FEW: 1 - 10, MODERATE: 11 - 49, HEAVY: >= 50

  **檢驗項目:Bacterial Culture(Ordinary) 檢體:PCN 報告日期:1081209**
  陰性結果: No growth for ordinary culture
  陽 菌
  性 落
  結 數
  果 目
  Ampicillin Cefazolin ClindamycinClarithromyGentamicin Amikacin Chloromycin
  (1)
  (2)
  (3)
  (4)
  Unasyn Penicillin GM(120ug) SMX+TMP Vancomycin Oxacillin Cefpirome
  (1)
  (2)
  (3)
  (4)
  Carbapen(IPM)CefoperazonCefotaxime Ceftazidime Cefepime Ceftriaxone Flomoxef
  (1)
  (2)
  (3)
  (4)
  MetronidazoleCiprofloxacMinocyclineTeicoplanin Tazocin CefmetazoneAztreonam
  (1)
  (2)
  (3)
  (4)
  Tigecycline Amox./Clav.Cefoxitin Levofloxa.Carbapen(MPM) Erythromycin
  (1)
  (2)
  (3)
  (4)
  Moxifloxacin Linezolid Ertapenem
  (1)
  (2)
  (3)
  (4)

  **檢驗項目:Blood Culture 檢體:Blood 報告日期:1081212**
  陰性結果: No bacterial growth for 7 days.
  陽 菌
  性 落
  結 數
  果 目
  Ampicillin Cefazolin ClindamycinClarithromyGentamicin Amikacin Chloromycin
  (1)
  (2)
  (3)
  (4)
  Unasyn Penicillin GM(120ug) SMX+TMP Vancomycin Oxacillin Cefpirome
  (1)
  (2)
  (3)
  (4)
  Carbapen(IPM)CefoperazonCefotaxime Ceftazidime Cefepime Ceftriaxone Flomoxef
  (1)
  (2)
  (3)
  (4)
  MetronidazoleCiprofloxacMinocyclineTeicoplanin Tazocin CefmetazoneAztreonam
  (1)
  (2)
  (3)
  (4)
  Tigecycline Amox./Clav.Cefoxitin Levofloxa.Carbapen(MPM) Erythromycin
  (1)
  (2)
  (3)
  (4)
  Moxifloxacin Linezolid Ertapenem
  (1)
  (2)
  (3)
  (4)

  **檢驗項目:Bacterial Culture(Anaerobic) 檢體:Drainage(Left penrose) 報告日期:1081202**
  陰性結果: No growth for anaerobic culture.
  陽 菌
  性 落
  結 數
  果 目
  Ampicillin Cefazolin ClindamycinClarithromyGentamicin Amikacin Chloromycin
  (1)
  (2)
  (3)
  (4)
  Unasyn Penicillin GM(120ug) SMX+TMP Vancomycin Oxacillin Cefpirome
  (1)
  (2)
  (3)
  (4)
  Carbapen(IPM)CefoperazonCefotaxime Ceftazidime Cefepime Ceftriaxone Flomoxef
  (1)

- (2)
(3)
(4)
MetronidazoleCiprofloxacMinocyclineTeicoplanin Tazocin CefmetazoneAztreonam
(1)
(2)
(3)
(4)
Tigecycline Amox./Clav.Cefoxitin Levofloxa.Carbapen(MPM) Erythromycin
(1)
(2)
(3)
(4)
Moxifloxacin Linezolid Ertapenem
(1)
(2)
(3)
(4)

**檢驗項目 :Bacterial Culture(Ordinary) 檢體:Drainage(Left penrose) 報告日期 :1081128**
陰性結果 :
陽 Pseudomonas aeruginosa 菌 H
性 落
結 數
果 目
Ampicillin Cefazolin ClindamycinClarithromyGentamicin Amikacin Chloromycin
(1) S S
(2)
(3)
(4)
Unasyn Penicillin GM(120ug) SMX+TMP Vancomycin Oxacillin Cefpirome
(1)
(2)
(3)
(4)
Carbapen(IPM)CefoperazonCefotaxime Ceftazidime Cefepime Ceftriaxone Flomoxef
(1) S S S S
(2)
(3)
(4)
MetronidazoleCiprofloxacMinocyclineTeicoplanin Tazocin CefmetazoneAztreonam
(1) S S
(2)
(3)
(4)
Tigecycline Amox./Clav.Cefoxitin Levofloxa.Carbapen(MPM) Erythromycin
(1)
(2)
(3)
(4)
Moxifloxacin Linezolid Ertapenem
(1)
(2)
(3)
(4)

**檢驗項目 :Bacterial Culture(Ordinary) 檢體:PCN 報告日期 :1081128**
陰性結果 : No growth for ordinary culture
陽 菌
性 落
結 數
果 目
Ampicillin Cefazolin ClindamycinClarithromyGentamicin Amikacin Chloromycin
(1)
(2)
(3)
(4)
Unasyn Penicillin GM(120ug) SMX+TMP Vancomycin Oxacillin Cefpirome
(1)
(2)
(3)
(4)
Carbapen(IPM)CefoperazonCefotaxime Ceftazidime Cefepime Ceftriaxone Flomoxef
(1)
(2)
(3)
(4)
MetronidazoleCiprofloxacMinocyclineTeicoplanin Tazocin CefmetazoneAztreonam
(1)
(2)
(3)
(4)
Tigecycline Amox./Clav.Cefoxitin Levofloxa.Carbapen(MPM) Erythromycin
(1)
(2)
(3)
(4)
Moxifloxacin Linezolid Ertapenem
(1)
(2)
(3)
(4)

**檢驗項目 :Bacterial Culture(Ordinary) 檢體:Sputum 報告日期 :1081127**
陰性結果 : Normal mixed flora presented.
陽 菌
性 落
結 數
果 目
Ampicillin Cefazolin ClindamycinClarithromyGentamicin Amikacin Chloromycin
(1)
(2)
(3)
(4)
Unasyn Penicillin GM(120ug) SMX+TMP Vancomycin Oxacillin Cefpirome
(1)
(2)
(3)
(4)
Carbapen(IPM)CefoperazonCefotaxime Ceftazidime Cefepime Ceftriaxone Flomoxef
(1)
(2)
(3)

- (4)
MetronidazoleCiprofloxacMinocyclineTeicoplanin Tazocin CefmetazoneAztreonam
(1)
(2)
(3)
(4)
Tigecycline Amox./Clav.Cefoxitin Levofloxa.Carbapen(MPM) Erythromycin
(1)
(2)
(3)
(4)
Moxifloxacin Linezolid Ertapenem
(1)
(2)
(3)
(4)

檢驗項目 :**GRAM STAIN** 檢體:**Sputum** 報告日期 :**1081126**
1) PMN >25 /LPF
2) Squamous epithelial cell 25 /LPF
3) Few Gram postive bacilli
@ SPUTUM: FEW: 1 - 10, MODERATE: 11 - 49, HEAVY: >= 50

檢驗項目 :**Blood Culture** 檢體:**Blood** 報告日期 :**1081201**
陰性結果： No bacterial growth for 7 days.
陽 菌
性 落
結 數
果 目
Ampicillin Cefazolin ClindamycinClarithromyGentamicin Amikacin Chloromycin
(1)
(2)
(3)
(4)
Unasyn Penicillin GM(120ug) SMX+TMP Vancomycin Oxacillin Cefpirome
(1)
(2)
(3)
(4)
Carbapen(IPM)CefoperazonCefotaxime Ceftazidime Cefepime Ceftriaxone Flomoxef
(1)
(2)
(3)
(4)
MetronidazoleCiprofloxacMinocyclineTeicoplanin Tazocin CefmetazoneAztreonam
(1)
(2)
(3)
(4)
Tigecycline Amox./Clav.Cefoxitin Levofloxa.Carbapen(MPM) Erythromycin
(1)
(2)
(3)
(4)
Moxifloxacin Linezolid Ertapenem
(1)
(2)
(3)
(4)

檢驗項目 :**Bacterial Culture(Ordinary)** 檢體:**Urine-indwelling catheter** 報告日期 :**1081127**
陰性結果： No growth for ordinary culture
陽 菌
性 落
結 數
果 目
Ampicillin Cefazolin ClindamycinClarithromyGentamicin Amikacin Chloromycin
(1)
(2)
(3)
(4)
Unasyn Penicillin GM(120ug) SMX+TMP Vancomycin Oxacillin Cefpirome
(1)
(2)
(3)
(4)
Carbapen(IPM)CefoperazonCefotaxime Ceftazidime Cefepime Ceftriaxone Flomoxef
(1)
(2)
(3)
(4)
MetronidazoleCiprofloxacMinocyclineTeicoplanin Tazocin CefmetazoneAztreonam
(1)
(2)
(3)
(4)
Tigecycline Amox./Clav.Cefoxitin Levofloxa.Carbapen(MPM) Erythromycin
(1)
(2)
(3)
(4)
Moxifloxacin Linezolid Ertapenem
(1)
(2)
(3)
(4)

檢驗項目 :**Blood Culture** 檢體:**Blood** 報告日期 :**1081201**
陰性結果： No bacterial growth for 7 days.
陽 菌
性 落
結 數
果 目
Ampicillin Cefazolin ClindamycinClarithromyGentamicin Amikacin Chloromycin
(1)
(2)
(3)
(4)
Unasyn Penicillin GM(120ug) SMX+TMP Vancomycin Oxacillin Cefpirome
(1)
(2)
(3)
(4)

- Carbapen(IPM)CefoperazonCefotaxime Ceftazidime Cefepime Ceftriaxone Flomoxef
  (1)
  (2)
  (3)
  (4)
  MetronidazoleCiprofloxacMinocyclineTeicoplanin Tazocin CefmetazoneAztreonam
  (1)
  (2)
  (3)
  (4)
  Tigecycline Amox./Clav.Cefoxitin Levofloxa.Carbapen(MPM) Erythromycin
  (1)
  (2)
  (3)
  (4)
  Moxifloxacin Linezolid Ertapenem
  (1)
  (2)
  (3)
  (4)

檢驗項目 :**Blood Culture** 檢體:**Blood** 報告日期 :**1081130**
陰性結果： No bacterial growth for 7 days.
陽性結果 菌落數目
Ampicillin Cefazolin ClindamycinClarithromyGentamicin Amikacin Chloromycin
(1)
(2)
(3)
(4)
Unasyn Penicillin GM(120ug) SMX+TMP Vancomycin Oxacillin Cefpirome
(1)
(2)
(3)
(4)
Carbapen(IPM)CefoperazonCefotaxime Ceftazidime Cefepime Ceftriaxone Flomoxef
(1)
(2)
(3)
(4)
MetronidazoleCiprofloxacMinocyclineTeicoplanin Tazocin CefmetazoneAztreonam
(1)
(2)
(3)
(4)
Tigecycline Amox./Clav.Cefoxitin Levofloxa.Carbapen(MPM) Erythromycin
(1)
(2)
(3)
(4)
Moxifloxacin Linezolid Ertapenem
(1)
(2)
(3)
(4)

檢驗項目 :**Blood Culture** 檢體:**Blood** 報告日期 :**1081130**
陰性結果： No bacterial growth for 7 days.
陽性結果 菌落數目
Ampicillin Cefazolin ClindamycinClarithromyGentamicin Amikacin Chloromycin
(1)
(2)
(3)
(4)
Unasyn Penicillin GM(120ug) SMX+TMP Vancomycin Oxacillin Cefpirome
(1)
(2)
(3)
(4)
Carbapen(IPM)CefoperazonCefotaxime Ceftazidime Cefepime Ceftriaxone Flomoxef
(1)
(2)
(3)
(4)
MetronidazoleCiprofloxacMinocyclineTeicoplanin Tazocin CefmetazoneAztreonam
(1)
(2)
(3)
(4)
Tigecycline Amox./Clav.Cefoxitin Levofloxa.Carbapen(MPM) Erythromycin
(1)
(2)
(3)
(4)
Moxifloxacin Linezolid Ertapenem
(1)
(2)
(3)
(4)

檢驗項目 :**Bacterial Culture(Ordinary)** 檢體:**Urine-voided midstream** 報告日期 :**1081126**
陰性結果：
陽性結果 Gram negative bacillus 菌落數目 1000
Ampicillin Cefazolin ClindamycinClarithromyGentamicin Amikacin Chloromycin
(1)
(2)
(3)
(4)
Unasyn Penicillin GM(120ug) SMX+TMP Vancomycin Oxacillin Cefpirome
(1)
(2)
(3)
(4)
Carbapen(IPM)CefoperazonCefotaxime Ceftazidime Cefepime Ceftriaxone Flomoxef
(1)

- (2)
(3)
(4)
MetronidazoleCiprofloxacMinocyclineTeicoplanin Tazocin CefmetazoneAztreonam
(1)
(2)
(3)
(4)
Tigecycline Amox./Clav.Cefoxitin Levofloxa.Carbapen(MPM) Erythromycin
(1)
(2)
(3)
(4)
Moxifloxacin Linezolid Ertapenem
(1)
(2)
(3)
(4)

**檢驗項目 :Bacterial Culture(Anaerobic) 檢體:Discharge(LEFT PENROSE) 報告日期 :1081127**
陰性結果： No growth for anaerobic culture.
陽性結果 菌落數目
Ampicillin Cefazolin ClindamycinClarithromyGentamicin Amikacin Chloromycin
(1)
(2)
(3)
(4)
Unasyn Penicillin GM(120ug) SMX+TMP Vancomycin Oxacillin Cefpirome
(1)
(2)
(3)
(4)
Carbapen(IPM)CefoperazonCefotaxime Ceftazidime Cefepime Ceftriaxone Flomoxef
(1)
(2)
(3)
(4)
MetronidazoleCiprofloxacMinocyclineTeicoplanin Tazocin CefmetazoneAztreonam
(1)
(2)
(3)
(4)
Tigecycline Amox./Clav.Cefoxitin Levofloxa.Carbapen(MPM) Erythromycin
(1)
(2)
(3)
(4)
Moxifloxacin Linezolid Ertapenem
(1)
(2)
(3)
(4)

**檢驗項目 :Bacterial Culture(Ordinary) 檢體:Discharge(LEFT PENROSE) 報告日期 :1081126**
陰性結果：
陽性結果 Pseudomonas aeruginosa 菌落數目 H
Ampicillin Cefazolin ClindamycinClarithromyGentamicin Amikacin Chloromycin
(1) S S
(2)
(3)
(4)
Unasyn Penicillin GM(120ug) SMX+TMP Vancomycin Oxacillin Cefpirome
(1)
(2)
(3)
(4)
Carbapen(IPM)CefoperazonCefotaxime Ceftazidime Cefepime Ceftriaxone Flomoxef
(1) S S S S
(2)
(3)
(4)
MetronidazoleCiprofloxacMinocyclineTeicoplanin Tazocin CefmetazoneAztreonam
(1) S S
(2)
(3)
(4)
Tigecycline Amox./Clav.Cefoxitin Levofloxa.Carbapen(MPM) Erythromycin
(1)
(2)
(3)
(4)
Moxifloxacin Linezolid Ertapenem
(1)
(2)
(3)
(4)

**檢驗項目 :Bacterial Culture(Anaerobic) 檢體:Discharge(ABD WUOND) 報告日期 :1081126**
陰性結果： No growth for anaerobic culture.
陽性結果 菌落數目
Ampicillin Cefazolin ClindamycinClarithromyGentamicin Amikacin Chloromycin
(1)
(2)
(3)
(4)
Unasyn Penicillin GM(120ug) SMX+TMP Vancomycin Oxacillin Cefpirome
(1)
(2)
(3)
(4)
Carbapen(IPM)CefoperazonCefotaxime Ceftazidime Cefepime Ceftriaxone Flomoxef
(1)
(2)
(3)

- (4)
  MetronidazoleCiprofloxacMinocyclineTeicoplanin Tazocin CefmetazoneAztreonam
  (1)
  (2)
  (3)
  (4)
  Tigecycline Amox./Clav.Cefoxitin Levofloxa.Carbapen(MPM) Erythromycin
  (1)
  (2)
  (3)
  (4)
  Moxifloxacin Linezolid Ertapenem
  (1)
  (2)
  (3)
  (4)

**檢驗項目 :Bacterial Culture(Ordinary) 檢體:Discharge(ABD WUOND) 報告日期 :1081123**
陰性結果：
陽 Pseudomonas aeruginosa 菌 H
性 Escherichia coli 落 H
結 數
果 目
Ampicillin Cefazolin ClindamycinClarithromyGentamicin Amikacin Chloromycin
(1) S S
(2) I=16 R>=16 S=2
(3)
(4)
Unasyn Penicillin GM(120ug) SMX+TMP Vancomycin Oxacillin Cefpirome
(1)
(2) R>=32 R>=320
(3)
(4)
Carbapen(IPM)CefoperazonCefotaxime Ceftazidime Cefepime Ceftriaxone Flomoxef
(1) S S S S
(2) S=0.5 S=8 S=0.12 S=0.12 S=0.25 S=2
(3)
(4)
MetronidazoleCiprofloxacMinocyclineTeicoplanin Tazocin CefmetazoneAztreonam
(1) S S
(2) S=0.25 S=4
(3)
(4)
Tigecycline Amox./Clav.Cefoxitin Levofloxa.Carbapen(MPM) Erythromycin
(1)
(2) S=4
(3)
(4)
Moxifloxacin Linezolid Ertapenem
(1)
(2) S=0.12
(3)
(4)

**檢驗項目 :Bacterial Culture(Ordinary) 檢體:Urine-straight catheter 報告日期 :1081116**
陰性結果：
陽 Pseudomonas aeruginosa 菌 >10000
性 落
結 數
果 目
Ampicillin Cefazolin ClindamycinClarithromyGentamicin Amikacin Chloromycin
(1) S S
(2)
(3)
(4)
Unasyn Penicillin GM(120ug) SMX+TMP Vancomycin Oxacillin Cefpirome
(1)
(2)
(3)
(4)
Carbapen(IPM)CefoperazonCefotaxime Ceftazidime Cefepime Ceftriaxone Flomoxef
(1) S S S S
(2)
(3)
(4)
MetronidazoleCiprofloxacMinocyclineTeicoplanin Tazocin CefmetazoneAztreonam
(1) S S
(2)
(3)
(4)
Tigecycline Amox./Clav.Cefoxitin Levofloxa.Carbapen(MPM) Erythromycin
(1)
(2)
(3)
(4)
Moxifloxacin Linezolid Ertapenem
(1)
(2)
(3)
(4)

**檢驗項目 :Stool Culture 檢體:Stool 報告日期 :1081031**
陰性結果： Neg. for Salmonella, Shigella and Vibrio.
陽 菌
性 落
結 數
果 目
Ampicillin Cefazolin ClindamycinClarithromyGentamicin Amikacin Chloromycin
(1)
(2)
(3)
(4)
Unasyn Penicillin GM(120ug) SMX+TMP Vancomycin Oxacillin Cefpirome
(1)
(2)
(3)
(4)
Carbapen(IPM)CefoperazonCefotaxime Ceftazidime Cefepime Ceftriaxone Flomoxef
(1)
(2)
(3)
(4)
MetronidazoleCiprofloxacMinocyclineTeicoplanin Tazocin CefmetazoneAztreonam

- (1)
(2)
(3)
(4)
Tigecycline Amox./Clav.Cefoxitin Levofloxa.Carbapen(MPM) Erythromycin
(1)
(2)
(3)
(4)
Moxifloxacin Linezolid Ertapenem
(1)
(2)
(3)
(4)

檢驗項目 :**Bacterial Culture(Ordinary)** 檢體:**Urine-indwelling catheter** 報告日期 :**1081025**
陰性結果 : No growth for ordinary culture.
陽 菌
性 落
結 數
果 目
Ampicillin Cefazolin ClindamycinClarithromyGentamicin Amikacin Chloromycin
(1)
(2)
(3)
(4)
Unasyn Penicillin GM(120ug) SMX+TMP Vancomycin Oxacillin Cefpirome
(1)
(2)
(3)
(4)
Carbapen(IPM)CefoperazonCefotaxime Ceftazidime Cefepime Ceftriaxone Flomoxef
(1)
(2)
(3)
(4)
MetronidazoleCiprofloxacMinocyclineTeicoplanin Tazocin CefmetazoneAztreonam
(1)
(2)
(3)
(4)
Tigecycline Amox./Clav.Cefoxitin Levofloxa.Carbapen(MPM) Erythromycin
(1)
(2)
(3)
(4)
Moxifloxacin Linezolid Ertapenem
(1)
(2)
(3)
(4)

檢驗項目 :**Blood Culture** 檢體:**Blood** 報告日期 :**1081029**
陰性結果 : No bacterial growth for 7 days.
陽 菌
性 落
結 數
果 目
Ampicillin Cefazolin ClindamycinClarithromyGentamicin Amikacin Chloromycin
(1)
(2)
(3)
(4)
Unasyn Penicillin GM(120ug) SMX+TMP Vancomycin Oxacillin Cefpirome
(1)
(2)
(3)
(4)
Carbapen(IPM)CefoperazonCefotaxime Ceftazidime Cefepime Ceftriaxone Flomoxef
(1)
(2)
(3)
(4)
MetronidazoleCiprofloxacMinocyclineTeicoplanin Tazocin CefmetazoneAztreonam
(1)
(2)
(3)
(4)
Tigecycline Amox./Clav.Cefoxitin Levofloxa.Carbapen(MPM) Erythromycin
(1)
(2)
(3)
(4)
Moxifloxacin Linezolid Ertapenem
(1)
(2)
(3)
(4)

檢驗項目 :**Blood Culture** 檢體:**Blood** 報告日期 :**1081029**
陰性結果 : No bacterial growth for 7 days.
陽 菌
性 落
結 數
果 目
Ampicillin Cefazolin ClindamycinClarithromyGentamicin Amikacin Chloromycin
(1)
(2)
(3)
(4)
Unasyn Penicillin GM(120ug) SMX+TMP Vancomycin Oxacillin Cefpirome
(1)
(2)
(3)
(4)
Carbapen(IPM)CefoperazonCefotaxime Ceftazidime Cefepime Ceftriaxone Flomoxef
(1)
(2)
(3)
(4)
MetronidazoleCiprofloxacMinocyclineTeicoplanin Tazocin CefmetazoneAztreonam
(1)
(2)

- (3)
  (4)
  Tigecycline Amox./Clav.Cefoxitin Levofloxa.Carbapen(MPM) Erythromycin
  (1)
  (2)
  (3)
  (4)
  Moxifloxacin Linezolid Ertapenem
  (1)
  (2)
  (3)
  (4)

  檢驗項目 :**Blood Culture** 檢體:**Blood** 報告日期 :**1081028**
  陰性結果 : No bacterial growth for 7 days.
  陽 菌
  性 落
  結 數
  果 目
  Ampicillin Cefazolin ClindamycinClarithromyGentamicin Amikacin Chloromycin
  (1)
  (2)
  (3)
  (4)
  Unasyn Penicillin GM(120ug) SMX+TMP Vancomycin Oxacillin Cefpirome
  (1)
  (2)
  (3)
  (4)
  Carbapen(IPM)CefoperazonCefotaxime Ceftazidime Cefepime Ceftriaxone Flomoxef
  (1)
  (2)
  (3)
  (4)
  MetronidazoleCiprofloxacMinocyclineTeicoplanin Tazocin CefmetazoneAztreonam
  (1)
  (2)
  (3)
  (4)
  Tigecycline Amox./Clav.Cefoxitin Levofloxa.Carbapen(MPM) Erythromycin
  (1)
  (2)
  (3)
  (4)
  Moxifloxacin Linezolid Ertapenem
  (1)
  (2)
  (3)
  (4)

---

## 病理報告

- 活體組織切片病理報告　手術日期：**108/10/22**　病理號：**S0837360**

  診斷醫師:陳韻女 報告日斯:108/10/24
  Pathologic diagnosis:
  Colon, endoscopic biopsy -— Adenocarcinoma, moderately
  differentiated.
  Gross description:
  The specimen consists of 4 pieces of tan soft tissue, up to
  0.8x0.4x0.1 cm. All for section.
  Microscopic description:
  Section shows colon mucosa with invasive carcinoma exhibiting
  glandular differentiation.
  Note:
  1. The case has been peer reviewed by two doctors.
  2. The diagnosis is concurred in intradepartment consensus meeting
  on 2019/10/24.
  #T-59300_2 #M-81403_2 2 1340 CM000F

  活體組織切片病理報告　手術日期：**108/11/06**　病理號：**S0839459**

  診斷醫師:江栾 報告日期:108/11/12
  Pathologic diagnosis:
  1. Ascending, transverse, and descending colon, terminal ileum, and
  appendix, extended right hemicolectomy -— a. Adenocarcinoma,
  moderately to poorly differentiated, of transverse colon, with
  extracellular mucin production. Tumor invades through the visceral
  peritoneum, and metastasizes to mesocolic lymph node (1/32). The
  appendix, ileocecal valve, and bilateral cut ends are free of tumor.
  b. Status post loop colostomy with stitch granuloma of mesocolon.
  2. Gallbladder, cholecystectomy -— Cholesterolosis.
  Ancillary study for diagnosis:
  IHC stains for MLH1, MSH2, PMS2 and MSH6 are done on section G. All
  above markers are preserved.
  Prognostic and predictive factor:
  1. Tumor size: 7.2x6.2x2 cm.
  2. Depth of invasion: Visceral peritoneum (including tumor
  continuous with serosal surface through area of inflammation).
  3. Histologic grade: G2-3: Moderately to poorly differentiated.
  4. Angiolymphatic invasion: Not identified.
  5. Perineural invasion: Not identified.
  6. Tumor deposits (discontinuous extramural extension): Not
  identified.
  7. Surgical margins:
  a) Proximal Margin: Uninvolved by invasive carcinoma, distance of
  tumor from margin: more than 100 mm.
  b) Distal Margin: Uninvolved by invasive carcinoma, distance of
  tumor from margin: 110 mm.
  c) Radial or Mesenteric Margin: Uninvolved by invasive carcinoma,
  distance of tumor from margin: more than 10 mm.
  8. Lymph node status: Involved (1/32).
  9. Extranodal involvement: Not identified.
  10. Tumor regression grading S/P CCRT: No known presurgical therapy.
  11. Type of polyp in which invasive carcinoma arose: Not identified.
  12. Additional pathologic findings: Not identified.
  13. Pathological TNM Stage: pT4aN1a (According to the eighth edition,
  2017, American Joint Committee on Cancer Staging Guidelines for
  Tumors / 2017 CAP guideline).
  14. TNM descriptors: Not applicable.
  Gross description:
  The specimen consists of 1) a segment of colon, 41 cm in length, and
  4 cm in proximal circumference, 6 cm in distal cut end circumference,
  and a segment of terminal ileum, 3.3 cm in length, and 4 cm in cut

- end circumference. An unremarkable appendix, 3.8 cm in length, is noted. A gray tan firm annular elevated and centrally ulcerative tumor, 7.2x6.2x2 cm, is noted, 11 cm to the distal cut end. On cross section, the tumor invades to the mesocolon with more than 10 mm to the radial margin and less than 1 mm to the serosa grossly. Serosal retraction is also noted focally. The appendix, ileocecal valve, and both cut ends are free of the tumor. 2) an opened gallbladder, a piece, 9x3.2x1.2 cm. The wall is gray tan soft and up to 0.3 cm in thickness. Neither stone nor tumor is found grossly. Representative part for section: A) gallbladder B) proximal cut end C) distal cut end D) appendix E) ileocecal valve F-K) tumor L-N) lymph nodes.
  Note:
  1. The interpretation of MMR panel and recommended studies are as below: (1) No loss of nuclear expression of MMR proteins: low probability of microsatellite instability-high (MSI-H). (2) Please note: There are exceptions to the above IHC interpretations. These results should not be considered in isolation, and clinical correlation with genetic counseling is recommended to assess the need for germline testing.
  2. This case has been peer reviewed by two doctors.
  #T-59441_2 #M-81403_2 3 1105 S0000F
  #T-C4520_2 #M-81406_2 3 1105 000000
  #T-59201_2 #M-09413_2 0 1105 000000

## 其它

## 出院時情況

- 3.改門診治療

## 出院指示

| 藥名 | 劑量 | 劑量單位 | 用法 | 頻次 | 數量 | 數量單位 | 現況 | 天數 | 單位 |
|---|---|---|---|---|---|---|---|---|---|
| Xanax tab 0.5mg | 1 | TAB | PO | HSPRN | 7 | TAB | TH | 7 | 天 |
| Norvasc tab 5mg | 1 | TAB | PO | BID | 14 | TAB | TH | 7 | 天 |
| 25mg Utapine tab | 1 | TAB | PO | QN | 7 | TAB | TH | 7 | 天 |
| Colchicine tab 0.5mg | 1 | TAB | PO | BID | 14 | TAB | TH | 7 | 天 |
| Nincort oral geL 6gm | | TUB | TOP | TID | 0 | TUB | TH | 1 | 瓶 |
| Cefaclor cap 250mg | 2 | CAP | PO | Q8H | 42 | CAP | TH | 7 | 天 |
| Ulstop tab 20mg | 1 | TAB | PO | BID | 14 | TAB | TH | 7 | 天 |

## 出院計劃

- 門診日期
  - date on 2019/12/25

主治醫師 2222H 林俊余 / 住院醫師 9981E 蔡采錚

正式摘要(電子簽章：**000000182315893)**

W55 / W55

doctorRank:B
